# Case Report: Paralytic ileus as an early manifestation of evolving neuroleptic malignant syndrome in a patient receiving anti-psychotics

**DOI:** 10.3389/fmed.2026.1911402

**Published:** 2026-09-03

**Authors:** Balakrishnan Arivalagan, Pragathi Munnangi, Yashkumar Girdharlal Kamani, Jyoti Guglani, Angel Barrera, Krishna Singh, Makker Jasbir, Harish Patel, Misbahuddin Khaja

**Affiliations:** 1 BronxCare Health System, New York, NY, United States; 2 Ross University School of Medicine, Bridgetown, Barbados

**Keywords:** atypical antipsychotics, bromocriptine, dantrolene, dopaminergic, paralytic ileus

## Abstract

Neuroleptic malignant syndrome (NMS) is an uncommon, but potentially life- threatening complication of antipsychotic therapy, primarily resulting from central dopaminergic blockade. NMS is a clinical diagnosis and occurs rarely in patients receiving atypical antipsychotics agents. Gastrointestinal manifestations, including severe hypomotility and paralytic ileus, are recognized adverse effects of antipsychotic medications. However, paralytic ileus preceding the characteristic neuromuscular and autonomic features of NMS is exceptionally rare and underrecognized as an early manifestation of the syndrome. We report the case of a 49-year-old man who presented with progressive abdominal distension secondary to intestinal pseudo-obstruction as an early manifestation of NMS. This atypical and underreported presentation highlights the importance of maintaining a high index of suspicion for NMS in patients receiving antipsychotic therapy who develop unexplained gastrointestinal dysmotility. Prompt recognition, immediate discontinuation of the offending antipsychotic agent, and early initiation of treatment with dantrolene and bromocriptine resulted in a favorable clinical outcome.

## Introduction

Neuroleptic malignant syndrome (NMS) is traditionally defined by a tetrad of hyperthermia, widespread muscle rigidity, altered mental status, and autonomic instability. The diagnosis of NMS is predominantly clinical, based on Levenson’s criteria. However, recognition can be difficult in the initial stages, especially when characteristic symptoms are absent, subtle, or delayed (1). Antipsychotic-induced dopaminergic blockade not only disrupts central thermoregulatory and motor pathways but also interferes with autonomic control of gastrointestinal motility through the gut–brain axis. Consequently, gastrointestinal hypomotility, including paralytic ileus, may occur. Nevertheless, paralytic ileus rarely represents the early presentation of NMS before the emergence of the characteristic signs of NMS. Such unusual presentations could create a diagnostic challenge, delay the management and may result in poor outcomes. We highlight the necessity for heightened vigilance in patients undergoing antipsychotic treatment who develop unexplained gastrointestinal dysmotility.

## Case presentation

**Figure 1:**
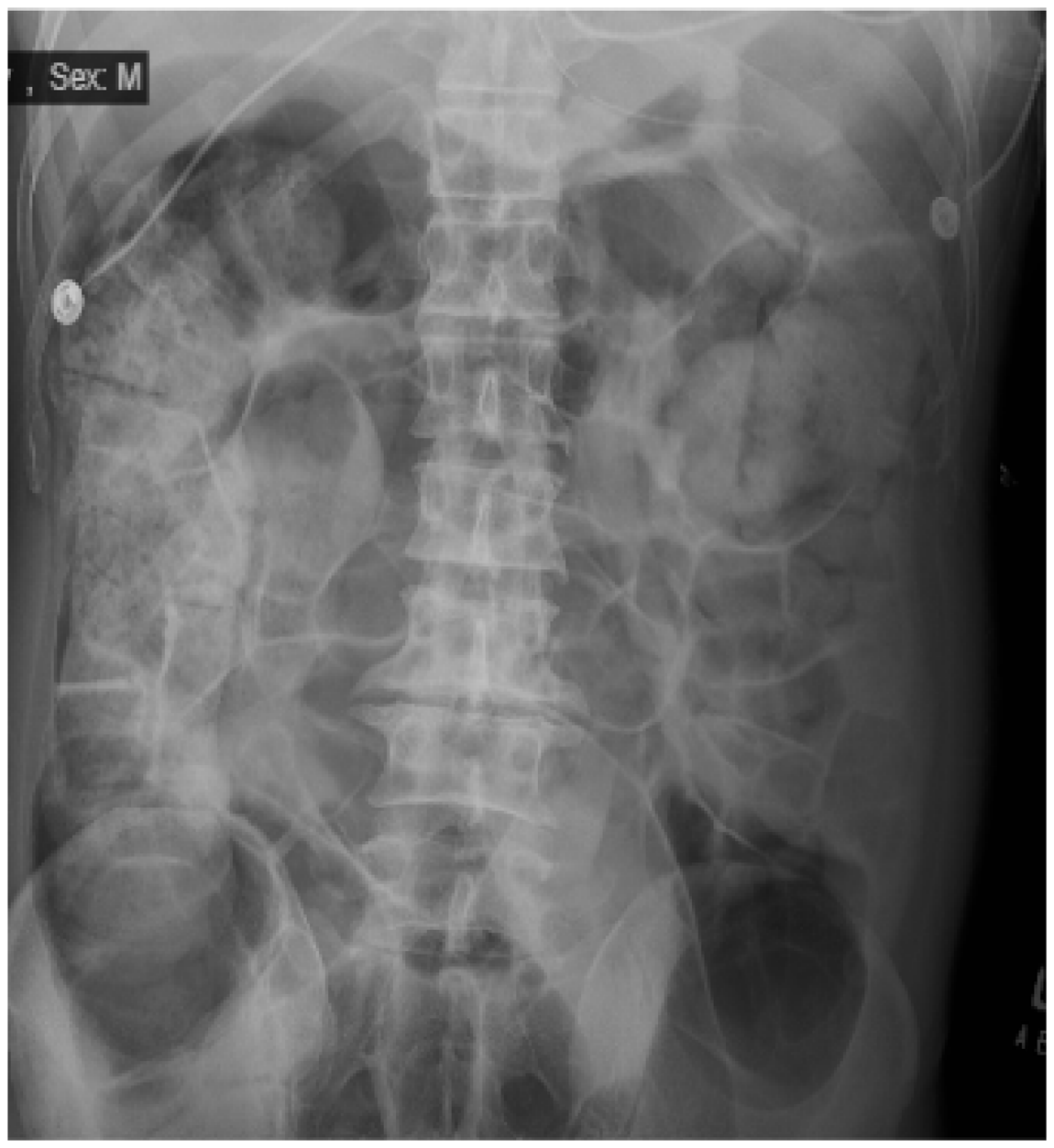
X ray abdomen on 6th day of admission shows dilated bowels with stool impaction without significant small obstruction or perforation.

**Figure 2:**
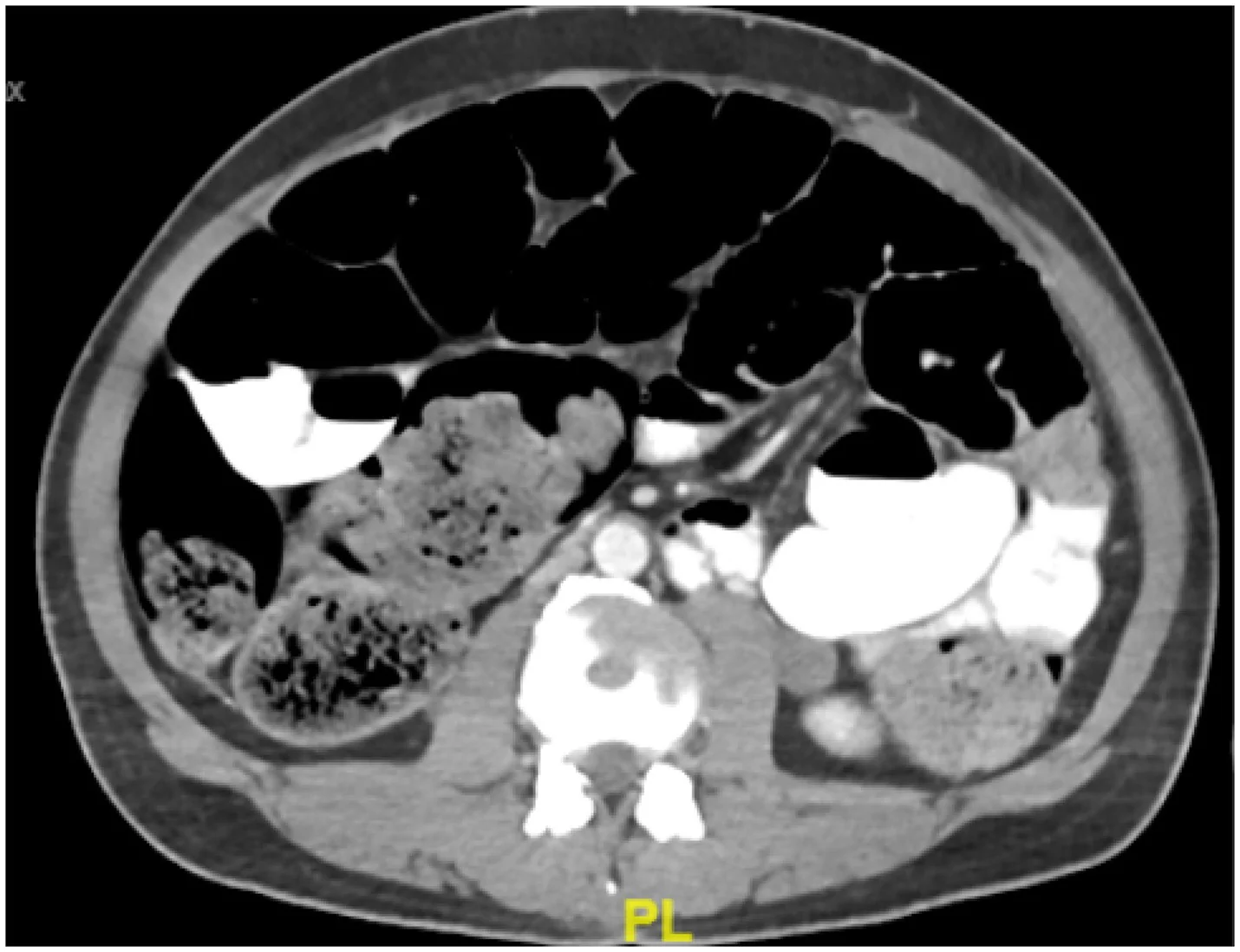
Contrast enhanced computed tomography (CECT) abdomen on 7th day of admission shows dilated bowels with stool impaction without significant small obstruction or perforation.

A forty-nine-year-old Hispanic male, with a medical history of schizoaffective disorder exhibited reduced verbal communication, diminished interaction, confusion, and increased distractibility since 14 Aug 2025. He was transferred from the inpatient psychiatry unit to the medical service for evaluation of an altered mental status August 17, 2025. He had been administered clozapine 225 mg twice daily, aripiprazole 30 mg at bedtime, and escitalopram 15 mg at bedtime for approximately one year without any alterations to the dosages. He received his last clozapine dose on August 17, 2025. He recognized his identity and location upon arrival but lacked awareness of the temporal context. His psychological condition hindered his ability to provide a comprehensive history. No evidence of fever or recent alterations in medication dosages. There were no concerns regarding substance abuse or withdrawal symptoms. He was afebrile, his heart rate was 88/min and blood pressure was 118/72 mmHg. There were no signs of meningism, focal neurological deficits, or muscular rigidity. The differential diagnoses were meningitis, encephalitis, metabolic disorders, and substance abuse. The computed tomography (CT) scan of head was unremarkable. The lumbar puncture revealed normal opening pressure, and the cerebrospinal fluid (CSF) analysis indicated no pleocytosis, had normal glucose and protein concentrations as mentioned in Table 1. Empirical intravenous antibiotics ceftriaxone and vancomycin was initiated for suspected occult infection. His blood, urine and CSF cultures were sterile, and urine toxicology was negative. On 24 Aug 2025, he developed abdominal distension and lack of bowel sounds. Abdominal radiography revealed significant distension of the intestinal loops as shown in Figure 1. A CT scan of the abdomen and pelvis revealed colon dilation, diffuse colon wall thickening, and stool impaction in the rectal vault as shown in Figure 2. There were no radiologic signs of pneumatosis intestinalis, free air, or bowel wall ischemia on serial abdominal imaging, and serum lactate levels remained within normal limits throughout evaluation. The abdominal examination showed no peritoneal signs, including guarding or rebound tenderness. Because the patient did not demonstrate features of toxic megacolon; such as severe systemic toxicity, marked colonic dilation with mucosal edema, or peritoneal irritation hence a surgical consultation was not pursued. A rectal tube was placed, and a watery, brownish stool was noted. Stool examinations, including ova, cyst and parasites and *Clostridium difficile* toxin assay, yielded negative results. On subsequent days, the patient showed marked confusion and exhibited rigidity across his entire body. He was febrile with a maximum temperature of 101.3 °F (38.5 °C). His blood pressure was erratic, accompanied by persistent tachycardia and diaphoresis. The pupils exhibited a uniform size and responded to light stimuli. The plantar responses were flexor bilaterally, and there was an absence of clonus and hyperreflexia. The infectious disease team was re-consulted and suspected colitis as the source of sepsis, prompting escalation to meropenem, yet the patient remained febrile despite intervention.

**Table 1:** Showing CSF analysis.

CSF analysis		
Parameter	Values	Reference values
CSF color	Colorless	Colorless
CSF appearance	Clear	Clear
RBC count	1.0	0–5/uL
WBC count	0.0	0–5/uL
Glucose	73	50–80 mg/dL
LDH	19	Less than 40 units/L
Protein	41	15–45 mg/dL
Bacterial Antigen	Negative	Negative
HSV 1 DNA	Not detected	Not detected
HSV 2 DNA	Not detected	Not detected
VDRL	Negative	Not detected
CMV	Not detected	Not detected

CSF, cerebrospinal fluid; RBC, red blood cell; WBC, White blood cell count; LDH, lactate dehydrogenase; HSV, herpes simplex virus; VDRL, Venereal Disease Research Laboratory; CMV, cytomegalovirus.

**Figure 3:**
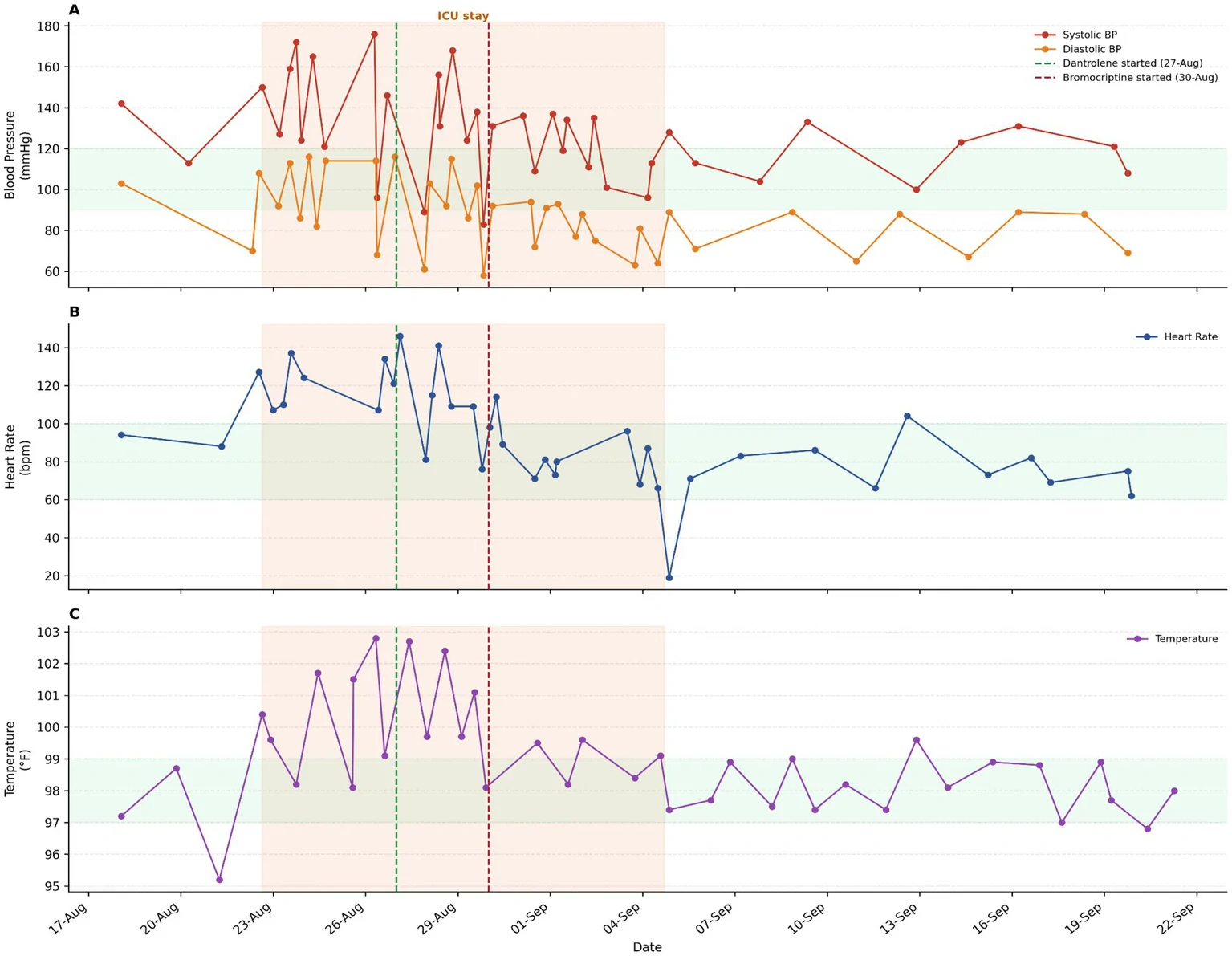
Showing serial trend in vital signs during hospitalization.

**Figure 4:**
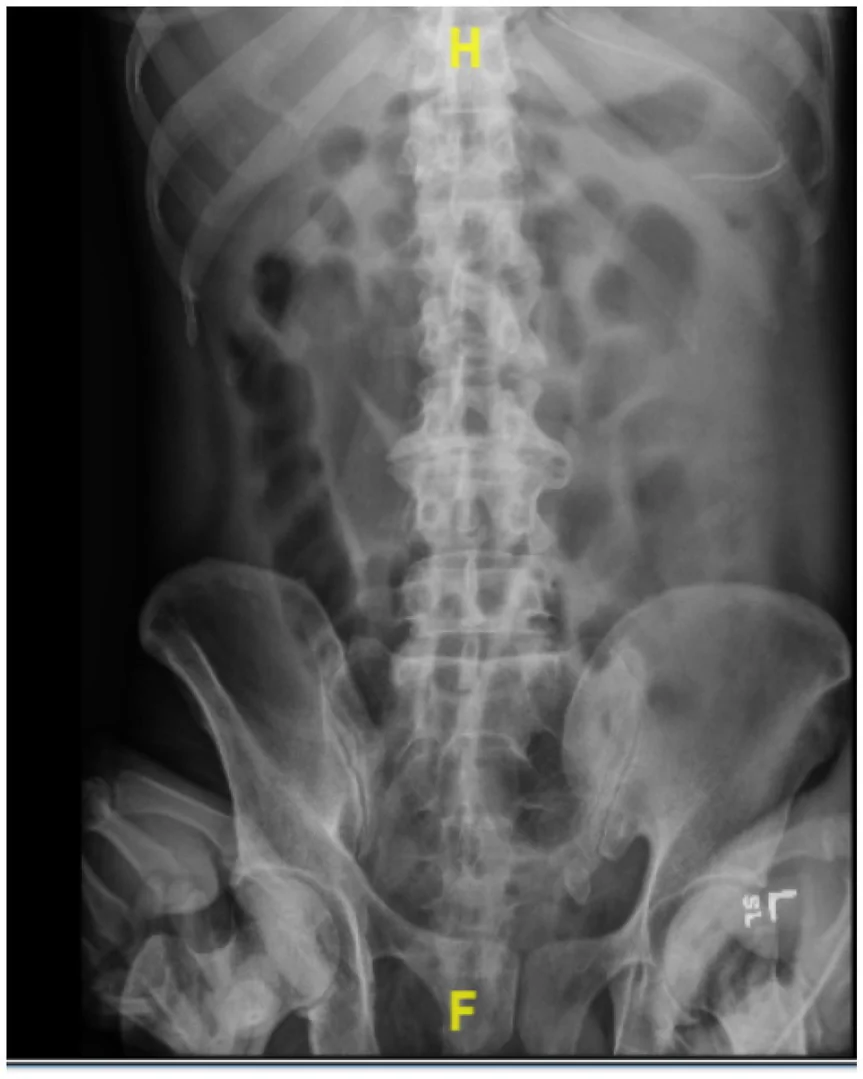
X ray abdomen on 3rd week of admission revealing normal bowel caliber in comparison with Figure 1.

Repeat blood, and urine cultures remained sterile, and further imaging and laboratory evaluations failed to identify an infectious origin. Neuroleptic malignant syndrome (NMS) was suspected due to the presence of hyperthermia, widespread rigidity, altered mental status, autonomic instability and simultaneous markedly elevated serum creatine phosphokinase (CPK) values of 55,000 IU/L (reference range: 20–200 IU/L). Based on Levenson’s major and minor criteria (shown in Table 2), the diagnosis of NMS was confirmed. We initiated administration of dantrolene at a dosage of 1 mg/kg loading dose and witnessed resolution of hyperthermia, rigidity and continued 1 mg/kg via intravenous infusion every 6 hours for 3 days (total cumulative dose of 280 mg/day), accompanied by rigorous supportive care. The patient had rapid clinical improvement, however, dantrolene was discontinued after 3 days due to elevated aspartate aminotransferase of 119 unit/L (reference range: 9–48 unit/L) and alanine aminotransferase of 69 unit/L (reference range: 5–40 unit/L). The next day, Bromocriptine 2.5 mg was administered via a nasogastric tube every 6 hours and continued for a total of 3 days followed by a three-day tapering period without adverse effects. In the initial week, his temperature, heart rate and blood pressure normalized as depicted in Figure 3. His white blood cell counts and creatine phosphokinase down trended as shown in Table 3. The patient became oriented and started verbalizing coherently by the second week of initiation of dantrolene and bromocriptine The paralytic ileus resolved in the fourth week of hospitalization, as confirmed by follow-up imaging studies, demonstrating a reduction in colonic diameter as shown in Figure 4. The patient required total of an eight-week hospitalization. He was medically and psychiatrically stable and discharged without any antipsychotics. He was advised close follow up with psychiatry as outpatient (Table 4).

**Table 2:** Showing Levenson’s criteria.

Major criteria	Noted in our patient
Fever	Yes
Rigidity	Yes
Elevated CPK levels	Yes

Minor criteria	Noted in our patient
Tachycardia	Yes
Fluctuating blood pressure	Yes
Leukocytosis	Yes
Altered mentation	Yes
Diaphoresis	Yes

**Table 3:** Showing trends of important labs.

Parameter	08/17	08/24	08/25	Dantrolene therapy			09/01	09/10	Reference values
				08/27	08/28	08/29			
WBC	10.1	5.26	4.59	7.7	9.2	11.4	10.0	5.3	4.8–10.8 k/ul
Platelet	289	290	256	240	346	395	407	432	150–400 k/ul
Na	139	145	154	154	151	145	144	140	135–145 mEq/L
K	4.5	4.0	4.2	4.4	4.1	3.8	3.6	4.8	3.5–5.0 mEq/L
Mg	2.5	2.2	2.5	2.8	2.6	2.3	2.4	2.5	1.5–2.7 mg/dL
Creatinine	0.8	0.8	0.9	1.0	0.6	0.5	0.4	0.6	0.5–1.5 mg/dL
Ca	9.6	8.6	8.2	7.8	8.1	8.3	8.3	9.1	8.5–10.5 mg/dL
CPK	234	294	1,062	3,685	5,470	986	346	220	20–200 unit/L
AST	21	18	42	81	109	119	35	16	9–48 unit/L
ALT	45	26	29	45	55	69	54	38	5–40 unit/L
ALP	75	77	63	50	52	47	63	99	56–119 unit/L
Special investigations									
Lactic acid	1.3	2.2	1.7	1.0	-	-	-	-	0.5–1.6 mmoles/L
Clozapine: 30, (Toxic range: Greater than 900 mcg/L) Norclozapine = 168 (25–400 mcg/L)									

Hgb, Hemoglobin; WBC, White blood cell count; Na, Sodium; K, Potassium; Mg, Magnesium; Ca, Calcium; CPK, Creatine phosphokinase; AST, Aspartate aminotransferase; ALT, Alanine aminotransferase; ALP, Alkaline phosphatase.

**Table 4:** Documented cases of paralytic ileus with NMS.

Citation	Age in years	Offending drug	Surgical management	Medical management	Outcome
Tanaka et al., 1993 (11)	48	Zotepine	None	Levodopa/Carbidopa for 3 weeks followed by Bromocriptine for 5 weeks	Discharge with complete resolution
Urvinget et al., 2003 (12)	73	Thioridazine and Paroxetine	Laparotomy on day of admission to relieve intestinal obstruction	Surgery followed by Bromocriptine given for 9 days	Discharged with complete resolution
Can SS et al., 2016 (13)	38	Paliperidone palmitate	Loop ileostomy operation	Recovered with surgery	Discharge with complete resolution
Campa et al., 2016 (14)	72	Levodopa and amantadine	None	Dantrolene 1 mg/kg for 3 days and calcium channel blockers	Cardiac arrest 9 days after admission due to refusal of dialysis
Chime et al., 2018 (15)	61	Trifluoperazine	Exploratory laparotomy	Post operative day 3 Dantrolene	Progressed to fatal asystole and death
Present case	42	clozapine	None	Dantrolene for 3 days followed by Bromocriptine for 6 days	Discharge with complete resolution

## Discussion

Epidemiologically, neuroleptic malignant syndrome occurs in only 0.02–3% of patients treated with dopamine-blocking agents, with first-generation antipsychotics such as haloperidol and fluphenazine being the most frequent triggers. The reported cases of NMS typically feature severe hyperthermia, autonomic instability, metabolic abnormalities, and marked elevations in muscle enzymes, with fever often refractory to standard antipyretics. Atypical antipsychotics including risperidone, olanzapine, and clozapine (as in this patient) have also been reported to cause NMS, but at lower rates. Notably, NMS rarely occurs in patients who have been on prolonged periods of antipsychotics with appropriate compliance as witnessed in this case (2, 3). The peculiar feature in this case is paralytic ileus presenting as an early manifestation of NMS, which is very uncommon. The pathophysiology of gastrointestinal dysfunction in NMS is complex. Blockade of central dopamine D2 receptors interferes with hypothalamic and basal ganglia function and thus disrupting the autonomic control of the gastrointestinal system besides causing increased muscle stiffness, high body temperatures, and significant changes in blood pressure (4, 5). Dopamine inhibits the release of acetylcholine, the primary excitatory neurotransmitter in the gut and modulates gastrointestinal motility.

Dysregulation of dopaminergic transmission, result in significant intestinal hypomotility and colonic dilatation. Additionally, rigid muscles and metabolic disorders may cause gastrointestinal problems even worse (6). These mechanisms elucidate the reasons why ileus may precede or obscure the conventional manifestations of NMS. The risk factors of ileus include electrolyte imbalance, dehydration, and other medications (opiods, anticholinergics or metoclopramide) were ruled out in the case. Gastrointestinal hypomotility is widely acknowledged as a significant consequence of antipsychotic medication, particularly with clozapine, which demonstrates strong anticholinergic and anti-serotonergic properties. Clozapine-induced gastrointestinal hypomotility (CIGH) has been linked to considerable morbidity and mortality, including constipation, paralytic ileus, bowel obstruction, ischemia, and perforation (7).

In this case, serum clozapine concentration of 301 mcg/L (toxic threshold of >900), and a norclozapine level of 168 mcg/L, (reference range of 25–400) were normal. The severity and persistence of ileus followed by the emergence of neurological and autonomic features suggest that the hypomotility represented not merely CIGH but the earliest manifestation of evolving neuroleptic malignant syndrome. It is also necessary to make a clinical differentiation with serotonin syndrome, especially in patients taking more than one psychotropic drug. According to Hunter Serotonin Toxicity Criteria, Serotonin syndrome usually shows up with hyperreflexia, ocular clonus and hyperactivity in the digestive system (like diarrhoea). In contrast, this patient had constipation and lacked hyperreflexia and clonus (8). Early diagnosis of NMS is essential, as delayed identification correlates with heightened morbidity, extended hospitalization, and the risk of sequelae including rhabdomyolysis, renal failure, and intestinal perforation. Management necessitates the immediate cessation of harmful agents, vigorous supportive care, and focused pharmacological intervention. Dantrolene, a skeletal muscle relaxant, lowers calcium release from the sarcoplasmic reticulum and improve stiffness and hyperthermia. Bromocriptine and other dopamine agonists work against central dopaminergic blockage (9, 10). For individuals with gastrointestinal involvement, it is prudent to correct electrolyte imbalances vigorously and be mindful about intestinal perforation. The individuals failing conservative therapy may require laparotomy for decompression or loop ileostomy. The available data suggests that delayed recognition of atypical NMS is associated with significant morbidity and mortality shown as Table 2 (11–15). In our case, the resolution of ileus coincided with systemic improvement following the initiation of dantrolene and bromocriptine, thereby substantiating NMS as the primary diagnosis.

## Conclusion

This case has significant implications for clinical practice. Firstly, ileus or pseudo- obstruction may manifest as an initial or presenting symptom of Neuroleptic Malignant Syndrome (NMS), prior to development of classic motor or systemic symptoms. Secondly, we emphasize that clinicians should not prematurely attribute severe gastrointestinal hypomotility solely to the side effects of antipsychotic therapy and need to rule out systemic crises. Finally, prompt diagnostic recognition and immediate discontinuation of the offending agent is critical to preventing fatal complications in atypical NMS presentations.

## Data Availability

The original contributions presented in the study are included in the article/supplementary material, further inquiries can be directed to the corresponding author.

## References

[1] Levenson JL. Neuroleptic malignant syndrome. Am J Psychiatry. (1985) 142:1137–45. doi: 10.1176/ajp.142.10.1137, 2863986

[2] Every-Palmer S Ellis PM. Clozapine-induced gastrointestinal Hypomotility: a 22-year bi-National Pharmacovigilance Study of serious or fatal slow gut reactions and comparison with international drug safety advice. CNS Drugs. (2017) 31:699–709. doi: 10.1007/s40263-017-0448-6, 28623627 PMC5533872

[3] De Hert M Hudyana H Dockx L Bernagie C Sweers K Tack J . Second-generation antipsychotics and constipation: a review of the literature. Eur Psychiatry. (2011) 26:34–44. doi: 10.1016/j.eurpsy.2010.03.003, 20542667

[4] Saunders MD. Acute colonic pseudo-obstruction. Best Pract Res Clin Gastroenterol. (2007) 21:671–87. doi: 10.1016/j.bpg.2007.03.001, 17643908

[5] Gurrera RJ. Sympathoadrenal hyperactivity and the etiology of neuroleptic malignant syndrome. Am J Psychiatry. (2011) 168:832–9. 10.1176/ajp.156.2.169 9989551

[6] Strawn JR Keck PE Jr Caroff SN. Neuroleptic malignant syndrome. Am J Psychiatry. (2007) 164:870–6. doi: 10.1176/ajp.2007.164.6.870, 17541044

[7] Every-Palmer S Nowitz M Stanley J Grant E Huthwaite M Dunn H . Clozapine-treated patients have marked gastrointestinal hypomotility, the probable basis of life-threatening gastrointestinal complications: a cross-sectional study. EBioMedicine. (2016) 5:125–34. doi: 10.1016/j.ebiom.2016.02.020, 27077119 PMC4816835

[8] Boyer EW Shannon M. The serotonin syndrome. N Engl J Med. (2005) 352:1112–20. doi: 10.1056/NEJMra041867, 15784664

[9] Velamoor VR. Neuroleptic malignant syndrome: a neuro-psychiatric emergency: recognition, prevention, and management. Asian J Psychiatr. (2017) 29:106–9. doi: 10.1016/j.ajp.2017.05.004, 29061403

[10] Rosenberg MR Green M. Neuroleptic malignant syndrome. N Engl J Med. (2022) 386:1641–52.

[11] Tanaka O Otani K Kondo T Kaneko S Fukushima Y. Paralytic ileus as a prodromal symptom of the neuroleptic malignant syndrome. Hum Psychopharmacol Clin Exp. (1993) 8:367–9. doi: 10.1002/hup.470080511

[12] Urving SH Nielsen EW. Neuroleptic malignant syndrome with severe liver failure. Acta Anaesthesiol Scand. (2003) 47:1041–3. doi: 10.1034/j.1399-6576.2003.00193.x, 12904200

[13] Can SS Kabadayı E. A case of ileus in a patient with schizophrenia under paliperidone palmitate treatment. Psychiatry Investig. (2016) 13:665–7. doi: 10.4306/pi.2016.13.6.665, 27909459 PMC5128356

[14] Campa D Ariello M Castaldo R Zibella F Ferrazza P Del Gaudio S. A fatal case of neuroleptic malignant syndrome after paralytic bowel in a patient taking antiparkinson medication. Eur J Case Rep Intern Med. (2016) 3:368. doi: 10.12890/2016_000368, 30755870 PMC6346863

[15] Chime C Alemam A Kumar K Dhallu M. Acute abdomen with ileus: a heralding presentation of neuroleptic malignant syndrome. Case Rep Gastroenterol. (2018) 12:566–9. doi: 10.1159/000492460, 30323731 PMC6180277

